# Blue rubber bleb nevus syndrome with concurrent esophageal carcinoma: A case report

**DOI:** 10.1097/MD.0000000000048523

**Published:** 2026-05-08

**Authors:** Sibin Mei, Yuewei Huang, Jing Liu, Qian Cao, Lingna Ye

**Affiliations:** a Department of Gastroenterology, Zhejiang University School of Medicine Affiliated Sir Run-Run Shaw Hospital, Hangzhou, China; b Zhejiang University School of Medicine, Hangzhou, China.

**Keywords:** blue rubber bleb nevus syndrome, esophageal squamous cell carcinoma, esophagectomy, esophagogastroduodenoscopy, vascular malformations

## Abstract

**Background:**

Blue rubber bleb nevus syndrome (BRBNS) is a hereditary disease characterized by venous malformation on the skin and throughout the gastrointestinal tract, which is usually considered a benign disease without elevated risk of carcinogenesis. Nevertheless, lumen narrowing caused by hemangiomas may result in repeated mucosal damage from mechanical stimuli such as solid food. Therefore, it is important to recognize the potential of malignancy as a result of such chronic inflammation due to partial obstruction.

**Case presentation:**

A 50-year-old patient presented with progressive dysphagia and fatigue. Physical examination showed bluish-violet and non-blanching blisters measuring 0.5 to 2 cm distributed over the skin. The patient has recurrent positive fecal occult blood tests 8 years ago and a low hemoglobin level 14 years ago. Chest computed tomography revealed obvious lumen wall thickening of the mid- and lower thoracic esophagus, accompanied by multiple nodular calcifications. Esophagogastroduodenoscopy and colonoscopy were performed to find multiple hemangiomas scattered throughout the GI tract, which is consistent with blue rubber bleb nevus syndrome (BRBNS). Additionally, biopsy of an ulcerated lesion in the esophageal lumen confirmed esophageal carcinoma. Subsequent positron emission tomography-computed tomography (PET-CT) and endoscopic ultrasound confirmed no metastasis. An esophagectomy was performed. At the 3-month follow-up, there was no evidence of recurrence.

**Conclusion:**

Currently, there are about 200 BRBNS cases reported, and none of these cases developed concurrent esophageal carcinoma. To our knowledge, this is the first case of BRBNS with concurrent esophageal carcinoma. Even though BRBNS is considered a benign disease, lumen narrowing caused by hemangiomas may result in repeated mucosal damage from mechanical irritation such as solid food, and physicians should be aware of the potential of malignancy due to chronic inflammation caused by partial obstruction.

## 1. Introduction

Blue rubber bleb nevus syndrome (BRBNS) is considered to be a rare disorder with about 200 to 300 cases reported in the medical literature to date.^[1]^ BRBNS consists mainly of abnormal blood vessels affecting the skin or internal organs, typically the gastrointestinal tract.^[2,3]^ It is characterized by multiple bluish-violet blebs on the skin, small bowel, and distal colon, which are usually present soon after birth. Cutaneous lesions can occur all over the skin, from the scalp to the feet, characterized by rubbery, telangiectatic, and soft lesions. Lesions can also present in the central nervous system, liver, and muscles. Numerous hemangiomas can cause bleeding and result in anemia.

The etiology is not completely understood. It may have an association with somatic mutations in the TEK (TIE2) gene and was first reported by William Bennett Bean in 1958, and therefore it is also termed as “Bean syndrome.”^[4]^ Patients may have a family history of multifocal venous malformations or recurrent gastrointestinal tract bleeding, even death from gastrointestinal bleeding. However, it can also present as a sporadic form without a significant family history.

Diagnosis is usually based on a combination of skin blebs and hemangiomas found in the gastrointestinal tract. Therefore, endoscopy should be performed to evaluate GI involvement upon suspicion of BRBNS.

There is no curative treatment for BRBNS so far. The management of BRBNS is categorized into conservative treatment, such as iron supplements and blood transfusions, and surgical interventions, such as laser ablation, electrocauterization, cryotherapy, and sclerotherapy. In recent years, mTOR inhibitor has been reported to not only reduce the hemangioma size throughout the gastrointestinal tract but also alleviate the gastrointestinal symptoms of patients.^[5-9]^ The majority of these cases were toddlers and teenagers, ranging from 15 months to 16 years old. A nationwide trial conducted in the Netherlands indicated that compared to adults, children had a higher response rate (93.8% vs 65.7%, *P* < .05) and faster response time (28 vs 91 days, *P* < .05) to sirolimus.^[10]^ However, Le Sage et al described no clinical benefit from topical sirolimus for verrucous venous malformation, since there is no lymphatic component in verrucous venous malformation.^[11]^ Surgical interventions are only necessary when patients present with anemia that requires repeated blood transfusions. Patients usually undergo entire or wedge resection of the affected GI segments, and up to 1-quarter of procedures need intraoperative endoscopic assistance. Clinical success was observed in all 29 patients undergoing surgery for BRBNS in a study.^[12]^ However, surgery is not curative, and lesions may recur after surgery.

Regular follow-up is highly recommended, including clinical evaluation, imaging, and laboratory tests. Certain circumstances such as puberty or pregnancy could promote the growth of lesions.

Some patients may complain about the recurrence of previously removed lesions after 5 to 10 years.^[13]^ Patients with advanced or recurrent disease require multidisciplinary therapy.

## 2. Case report

### 2.1. Patient information

A 50-year-old man presented to the clinic with progressive dysphagia and fatigue for 1 month. He has recurrent positive fecal occult blood tests 8 years ago and a low hemoglobin level 14 years ago.

### 2.2. Clinical findings

On physical examination, bluish-violet and non-blanching blisters measuring 0.5 to 2 cm were distributed over the skin, which had been present for >40 years, according to his statement. Laboratory studies showed a hemoglobin level of 11.7 g/dL, a hematocrit of 35.2%, and blood smear indicated microcytic hypochromic anemia.

### 2.3. Diagnostic assessment

**Figure 1.**
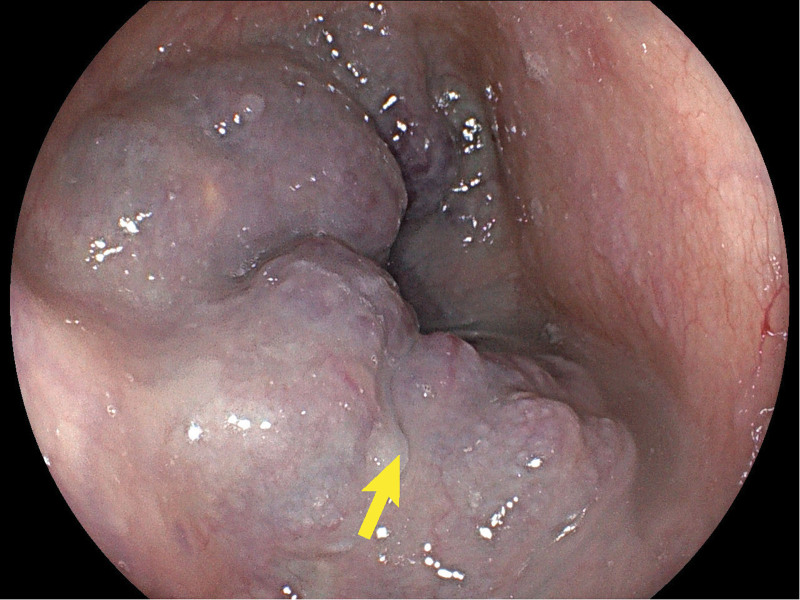
Multiple bluish-violet grape-like hemangiomas obstructed the esophageal lumen.

**Figure 2.**
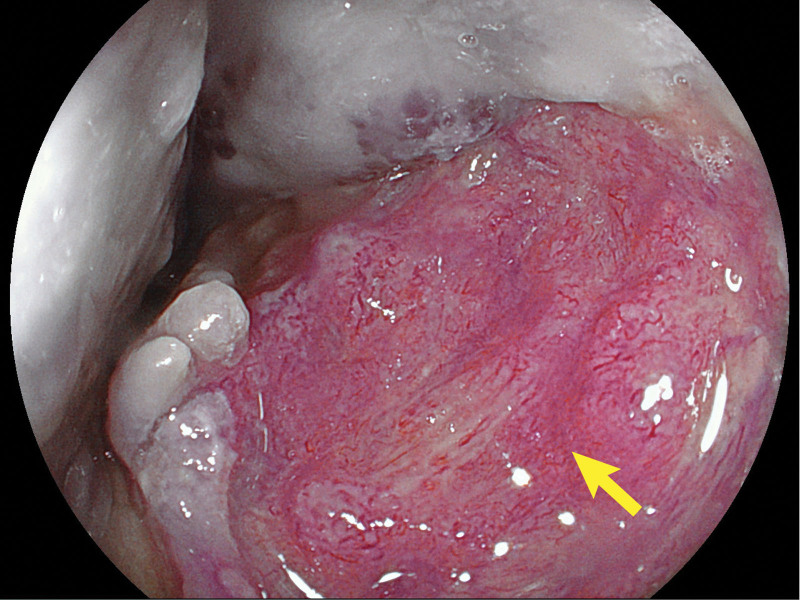
A large ulcerated lesion in esophageal lumen.

**Figure 3.**
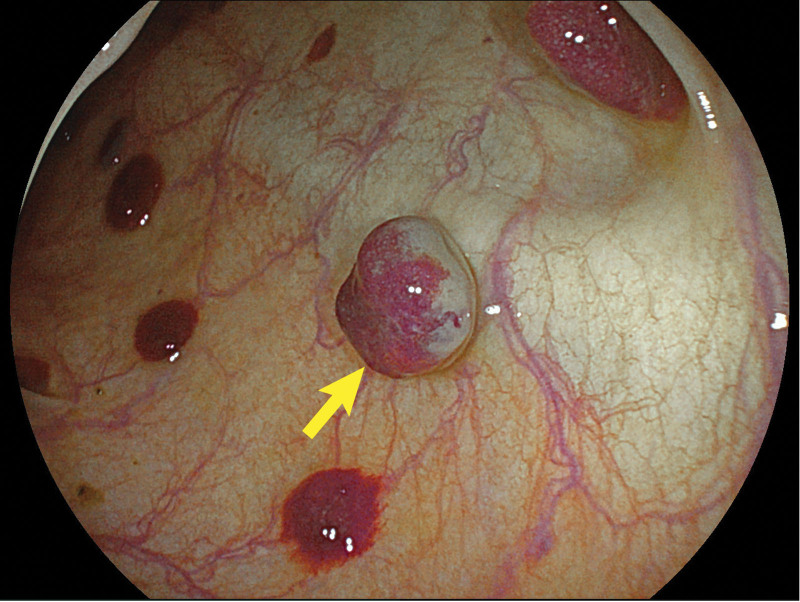
Hemangiomas scattered throughout the colon.

Chest computed tomography revealed obvious lumen wall thickening of the mid- and lower thoracic esophagus, accompanied by multiple nodular calcifications. Additionally, the adjacent left and right main bronchi were compressed. An esophagogastroduodenoscopy was performed. Esophagogastroduodenoscopy revealed diffuse, multiple bluish-violet hemangiomas 20 to 35 cm distal to the incisors, representing grape-like changes obstructing the esophageal lumen (Fig. 1). A large ulcerated lesion encircling one-third of the esophageal lumen was noted 27 to 32 cm distal to incisors (Fig. 2). The ulcer has an irregular morphology with raised margins and a necrotic base. The gross appearance of the ulcerated lesion raised the suspicion of cancer, so a biopsy was taken. Multiple bluish-violet hemangiomas of varying sizes were present in the gastric body, antrum, duodenal bulb, and descending part of the duodenum. Since the blebs and the ulcerated lesion were explored during the esophagogastroduodenoscopy, a further colonoscopy was warranted. Subsequent colonoscopy revealed numerous hemangiomas scattered throughout the colon (Fig. 3). An esophageal biopsy specimen was obtained from the ulcerated mucosa overlaying a large hemangioma with no chronic inflammation alongside epithelial dysplasia in the mucosal tissue on histopathological examination. A skin lesion biopsy was taken, which is consistent with hemangioma. Based on these findings, the patient was diagnosed with BRBNS and concurrent esophageal squamous cell carcinoma.

### 2.4. Therapeutic intervention

Upon diagnosis of carcinoma, a PET-CT and endoscopic ultrasound were performed with confirmed no distal metastasis. A subsequent tri-incisional (McKeown) esophagectomy was performed. The pathology of the surgical specimen confirmed squamous cell carcinoma without lymph node metastasis. Also, multiple hemangiomas in the submucosa and propria were observed, further supporting the diagnosis of BRBNS. Postoperative chemotherapy with paclitaxel and cisplatin was scheduled 2 months after surgery. The patient didn’t tolerate chemotherapy well. Soon after chemotherapy, the patient presented with neutropenia and a low hemoglobin level ranging from 50 to 70 mg/dL which can’t be corrected. So the chemotherapy was stopped. Currently, the patient is undergoing intermittent intravenous iron supplements with a stable hemoglobin of 80 mg/dL. Radiotherapy has not been initiated due to risk of bleeding from the hemangiomas.

### 2.5. Follow-up and outcomes

The patient is currently under close follow-up, and a recent esophagogastroduodenoscopy at the 3-month follow-up revealed no recurrence of the carcinoma.

The timeline of the patient has been listed in Table 1.

**Table 1:** Timeline of the test, diagnosis, surgery, and follow-up of the patient.

Presentation/abnormal test	Time
Low hemoglobin during annual physical examination	2011
Recurrent positive FOBTs	Since 2017
Progressive dysphagia and fatigue	March 2025
Diagnosis	April 10–17, 2025
CT scan	April 10, 2025
EGD endoscopy	April 11, 2025
Colonoscopy	April 11, 2025
Skin biopsy	April 14, 2025
MDT	April 16, 2025
PET-CT	April 17, 2025
Surgery	April 18, 2025
Esophagectomy	April 18, 2025
Follow-up	June 23, 2025, follow up at other hospital
Chemotherapy	June 23 2025

CT = computed tomography, PET-CT = positron emission tomography-computed tomography.

## 3. Discussion

For this patient, chronic bleeding leads to anemia and fatigue. Additionally, the patient presented with a recurrent positive fecal occult blood test. The explanation of the recurrent positive fecal occult blood test since 2017 is probably the chronic bleeding of the hemangiomas throughout the gastrointestinal tract and the newly developed cancer. Since BRBNS patients develop hemangiomas throughout the gastrointestinal tract since infancy, chronic physical irritation between the food bolus and hemangiomas results in bleeding. Therefore, we are not able to identify the specific region of bleeding. For newly diagnosed esophageal cancer, the physical irritation could probably result in bleeding.

Blue rubber bleb nevus syndrome is usually regarded as a benign disease with no significant increase in risk of neoplasia.^[2-4]^ Currently, there are about 200 BRBNS cases reported, and none of these cases reported the incidence of concurrent esophageal carcinoma.^[1]^ However, in this case report, the patient was diagnosed with BRBNS with concurrent esophageal squamous cell carcinoma developing overlying a large hemangioma, raising concerns about whether the 2 conditions could be related. To date, there are no reported cases or literature on BRBNS with concurrent esophageal carcinoma. While a direct genetic link between BRBNS and esophageal cancer is not established, we propose a plausible pathogenesis mediated by chronic mechanical irritation and inflammation. In general, esophageal squamous cell carcinoma is related to smoking, alcohol exposure, and thermal damage to the esophagus.^[14]^ Though mechanical irritation is not a direct risk factor for esophageal carcinoma, it can lead to esophageal mucosa injury and subsequent chronic inflammation, which potentially increases the risk of carcinoma. In fact, a previous study reported that physical injury due to very coarse food (silica fibers and millet bran) can predispose the patient to developing esophageal carcinoma.^[15]^ Irritation of the esophagus caused by high-fiber food should be avoided in patients with previous GERD.^[16]^ In this patient with hemangiomas obstructing the esophageal lumen, lumen narrowing predisposes the patient to recurrent mucosal injury from mechanical irritation by solid food. Persistent chronic inflammation promotes the release of inflammatory cytokines, thereby accelerating the process of cell growth and invasion, and thus the progression of carcinogenesis.^[17]^ Besides that, the patient had skin lesions soon after birth for >40 years, suggesting long-standing BRBNS. Chronic esophageal irritation expedites the development of premalignant transformation, resulting in subsequent cancer.

The treatment of choice for esophageal carcinoma is surgery, such as transhiatal esophagectomy, Ivor-Lewis transthoracic esophagectomy, and tri-incisional (McKeown) esophagectomy, followed by a combination of chemoradiotherapy. However, the concurrent BRBNS increases the complexity of management for this patient. First, the venous malformation increases the risk of bleeding, infection, and anastomotic leakage. Second, while esophageal squamous cell carcinoma is sensitive to radiotherapy,^[18]^ the effect of radiotherapy on extensive hemangiomas is unknown and could potentially increase the risk of bleeding or other complications. Furthermore, mTOR inhibitor is playing a more vital role in oncotherapy currently. Its antineoplastic properties could be beneficial theoretically to patients with cancer. According to previous studies, it is able to enhance the immune response to cancer and promote cancer regression in clinical trials. For example, temsirolimus (CCI-779) or everolimus (RAD001) are being tested for treatment in glioblastoma multiforme and mantle cell lymphoma.^[19]^ Additionally, mTOR inhibitors could block specific proliferative targets, promoting the effectiveness of other chemotherapy drugs like doxorubicin. For example, mTOR inhibitors block Akt signaling, which promotes cell survival and resistance to chemotherapy in Akt-positive lymphomas, thus promoting the effectiveness of chemotherapy. However, there is a lack of data regarding the use of mTOR inhibitors among immunocompromised patients undergoing chemotherapy, since mTOR inhibitors have been reported to cause severe adverse events, primarily infections or sepsis, in clinical research.^[20]^ In general, physicians should balance the risk of infection and the theoretical benefit of antineoplastic properties to decide whether to avoid mTOR inhibitor.

For this patient, although esophageal carcinoma has been essentially controlled and the BRBNS remains relatively stable, it remains critically important to maintain cautious management of the esophageal cancer while implementing close monitoring and follow-up for potential BRBNS-related complications such as hemorrhage.

Additionally, the raised hemangiomas can result in poor visual field and ignorance of carcinoma lesions during endoscopic procedure. It is important to note that the presence of multiple hemangiomas in the esophagus could obscure the visual field during the endoscopic procedure, making a diagnosis of concurrent malignancy harder, and physician should raise a high index of suspicion especially when the clinical symptoms changed rapidly.

In conclusion, this is the first reported case of BRBNS with concurrent esophageal carcinoma. To date, we are not able to establish a relationship between BRBNS and esophageal carcinoma. We aim to raise physicians’ awareness of this phenomenon to prevent potential ignorance of the possibility of tumors in such patients. Additionally, this case report provides a view to further exploration of the relationship.

## Acknowledgments

We thank Department of Pathology in Sir Run Run Shaw Hospital for assistance with the pathological report. We also wish to express our gratitude to the patient who participated in this study. This work was supported by Dr Lingna Ye’s medical team.

## Author contributions

**Conceptualization:** Sibin Mei, Jing Liu, Qian Cao, Lingna Ye.

**Data curation:** Sibin Mei, Yuewei Huang.

**Investigation:** Yuewei Huang.

**Methodology:** Sibin Mei, Jing Liu, Qian Cao, Lingna Ye.

**Supervision:** Sibin Mei, Jing Liu, Qian Cao, Lingna Ye.

**Visualization:** Yuewei Huang.

**Writing – original draft:** Sibin Mei, Yuewei Huang.

**Writing – review & editing:** Sibin Mei, Yuewei Huang, Jing Liu.
